# CdS Nanoplates Modification as a Platform for Synthesis of Blue-Emitting Nanoparticles

**DOI:** 10.3390/ijms22126477

**Published:** 2021-06-17

**Authors:** Anna Lesiak, Mateusz Banski, Hanna Woznica, Andrzej Żak, Joanna Cabaj, Artur Podhorodecki

**Affiliations:** 1 Faculty of Fundamental Problems of Technology, Wroclaw University of Science and Technology, Wybrzeze Wyspianskiego 27, 50–370 Wroclaw, Poland; anna.lesiak@pwr.edu.pl (A.L.); hanna.woznica@pwr.edu.pl (H.W.); artur.p.podhorodecki@pwr.edu.pl (A.P.); 2 Faculty of Chemistry, Wroclaw University of Science and Technology, Wybrzeze Wyspianskiego 27, 50–370 Wroclaw, Poland; joanna.cabaj@pwr.edu.pl; 3 Electron Microscopy Laboratory, Faculty of Mechanical Engineering, Wroclaw University of Science and Technology, Wybrzeze Wyspianskiego 27, 50–370 Wroclaw, Poland; andrzej.zak@pwr.edu.pl

**Keywords:** 3-mercaptopropionic acid, blue emitting quantum dots, d-penicillamine, ligand exchange, CdS nanoplatelets, phase transfer

## Abstract

In this paper, the study of surface modification of two-dimensional (2D), non-luminescent CdS nanoplates (NPLs) by thiol-containing ligands is presented. We show that a process of twophase transfers with appropriate ligand exchange transforms non-luminescent NPLs into spherical CdS nanoparticles (NPs) exhibiting a blue photoluminescence with exceptionally high quantum yield ~90%. In the process, transfer from inorganic solvent to water is performed, with appropriately selected ligand molecules and pH values (forward phase transfer), which produces NPs with modified size and shape. Then, in reverse phase transfer, NPs are transferred back to toluene due to surface modification by combined Cd (OL)_2_ and Cd (Ac)_2_. As a result, spherical NPs are formed (average diameter between 4 and 6 nm) with PL QY as high as 90%. This is unique for core only CdS NPs without inorganic shell.

## 1. Introduction

Colloidal nanoplates (NPLs) represent a type of two-dimensional (2D) semiconducting nanoparticle (NP) and possess a strong one-dimensional (1D) quantum confinement. Together with zero-dimensional quantum dots (QDs) and 1D nanorods (NRs), the NPLs complete the family of quantum-confined semiconductor NPs [1]. Each type of geometric structure has its own unique optical properties which can be controlled by the size of the NPs [2].

In the case of nanoplates, their optical properties are closely related to quantum confinement induced by their well-defined thickness equal few monolayers (ML) [3]. Due to this, NPLs have narrow photoluminescence bands (FWHM_PL_, 7–10 nm), ultrafast PL lifetime (τ_PL_ ~200–400 ps), and large absorption cross section (10^−14^ cm^2^) [4]. However, due to the shape and crystalline structure defined by surface ligand applied during synthesis, any post-synthesis modification of the NPLs surface should be carried out very carefully [5]. Surface energy change implied by ligand exchange may cause degradation and/or transformation of the NPLs into spherical structures or nanorods [6].

On the other hand, surface modifications of the NPs not only aim to improve their stability but are also responsible for new functions and properties of NPs [7]. In addition, NPs surface modification is necessary for phase transfer processes, i.e., the transfer of nanoparticles from the organic to the inorganic environment or conversely [8].

In our two previous papers [9,10], it has been shown that the pH at which ligand exchange reaction (LE) is conducted has a significant influence on the obtained NPs and their properties, i.e., colloidal and photoluminescence stability. Moreover, we also showed that after surface modification of CdS NPLs by d-penicillamine (DPA) coating, out of two peaks visible in the absorbance spectrum of hydrophobic CdS NPLs (375 nm and 410 nm), only one absorption peak remained at 402 nm for hydrophilic NPLs. It was assumed that due to LE, the bimodal distribution (5 and 6 monolayers) was converted to a single-mode distribution (5.5 monolayers, i.e., 5 ML od CdS finished by thiol group) [10,11].

This study presents two stages of NP surface modification. In the first stage, the surface modification of two types of NPLs was carried out by the exchange of post synthesis ligands on 3-mercaptopropionic acid (3-MPA) or d-penicillamine, in the acidic and alkaline environment. Ultimately, hydrophilic NPs were obtained and their optical properties were discussed. In the second stage, the reverse surface modification was carried out. The hydrophilic NPs obtained in the first step were treated by hydrophobic ligands and NPs became hydrophobic again. The experimental results were analysed and described as a new method of core NP synthesis having a highly efficient blue emission.

## 2. Results and Discussion

**Figure 1:**
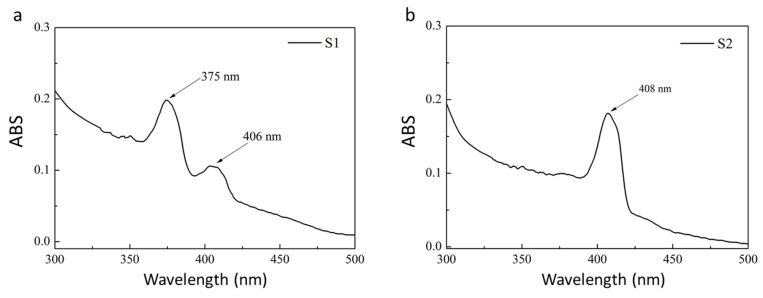
Absorbance spectra of CdS NPLs before LE: (**a**) sample S1; (**b**) sample S2.

**Figure 2:**
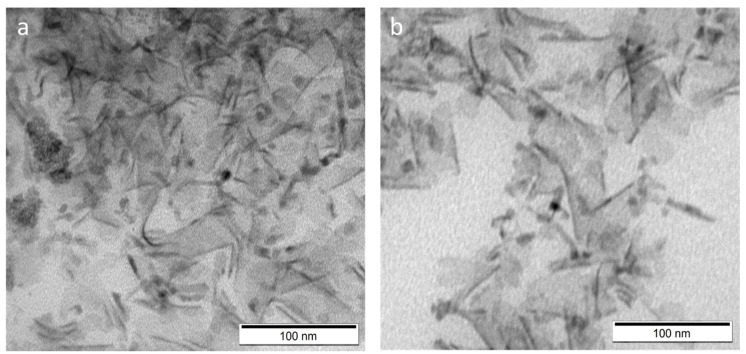
TEM image of CdS NPLs before LE: (**a**) sample S1; (**b**) sample S2.

Two types of NPLs were synthesized and their absorbance spectra are shown in Figure 1a,b. Sample S1 showed the formation of two types of NPLs with different thicknesses (5 monolayers (375 nm) and 6 monolayers (406 nm)), while sample S2 contained only one type of NPL thickness, 6 monolayers (408 nm) [11,12]. None of the samples showed photoluminescence. The structure of obtained NPLs was confirmed by TEM measurements (Figure 2a,b). The morphological presentation of NPLs showed that they are flat flakes with uneven edges of similar lateral sizes below 100 nm, however it is difficult to determine the unequivocal size of one nanoplate, due to its twisting. The very small thickness of NPLs may cause interactions between NPLs, as a result of which they are “rolling-up” [10].

In order to perform a phase transfer of NPLs, a ligand exchange procedure was proposed for both kinds of synthesized NPLs. Oleate ligands were replaced with 3-MPA or DPA, while PB (pH = 7.4) was used as an aqueous phase in both cases. Table 1 shows names of the samples and conditions of forward phase transfer.

**Figure 3:**
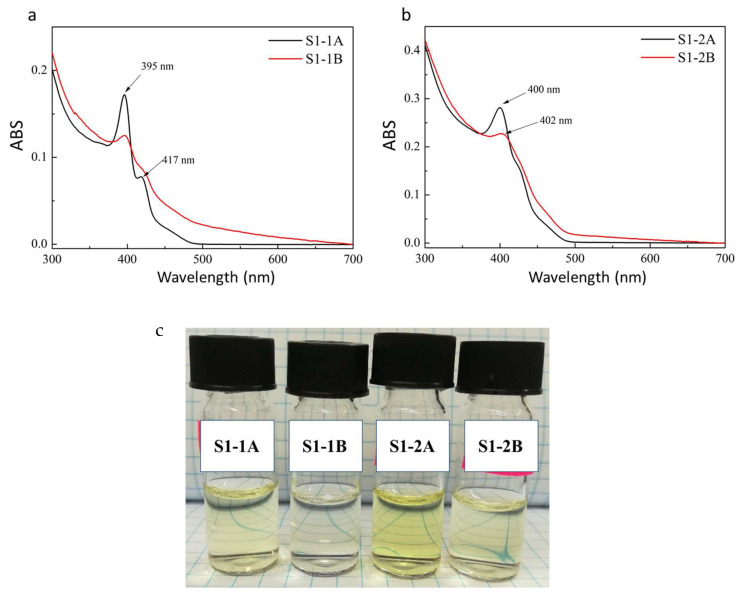
Absorbance spectra of NPLs after surface modification of sample S1 by (**a**) 3-MPA; (**b**) DPA ligand. LE conducted under acidic (black line) and alkaline (red line) conditions. (**c**) Digital image of NPLs solution dispersed in PB.

**Figure 4:**
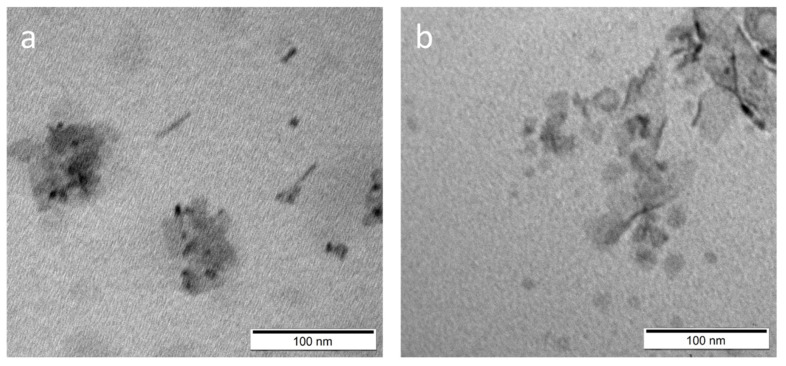
TEM pictures of NPLs (sample S1) after surface modification by (**a**) 3-MPA; (**b**) DPA ligand. Both samples have been modified in acidic conditions.

ABS measurements were performed after forward phase transfer and normalized results are shown in Figure 3a,b or sample S1. The two peaks derived originally from S1 NPLs (at 375 nm and 406 nm) changed to two sharp features at 395 nm and 417 nm after 3-MPA surface modification, while only one prominent peak shifted to 400 nm is present after DPA modification. For both ligands, the forward phase transfer proceeded with much better efficiency under acidic (samples S1-1A and S1-2A) than under alkaline (samples S1-1B and S1-2B) conditions. Based on the ABS results for the NPLs solution directly after forward phase transfer (without dilution), it was found that there is 5.5-times more nanoparticles in the S1-1A than in the S1-1B sample, and 3.7-times more NPLs was found in the S1-2A than in the S1-2B sample. This is clearly evidenced as a difference in the intensity of the sample colour (Figure 3c). Figure 4a,b shows TEM images for S1-1A and S1-2A, respectively. Both samples underwent surface etching [6]. The sample S1-1A shows the presence of structures of various dimensions; in addition to flat structures, both spherical and elongated nanoparticles are present. They show a tendency to aggregate. In the case of sample S1-2A, 2D nanoparticles like those presented for the starting sample S1 are still observed. They are in various size, smaller and larger than in the original S1 sample, but a lack of end-roll affinity was noticed. This may indicate that DPA as a ligand incorporated into the NPLs structures forming an additional half-monolayer. Absorbance peak at 400 nm corresponding to 3.1 eV is characteristic for 5.5 monolayers of CdS NPLs [13]. Additional half-monolayer caused the NPLs to be more rigid. The photoluminescence of the obtained samples was also measured, but none of them showed emission.

**Figure 5:**
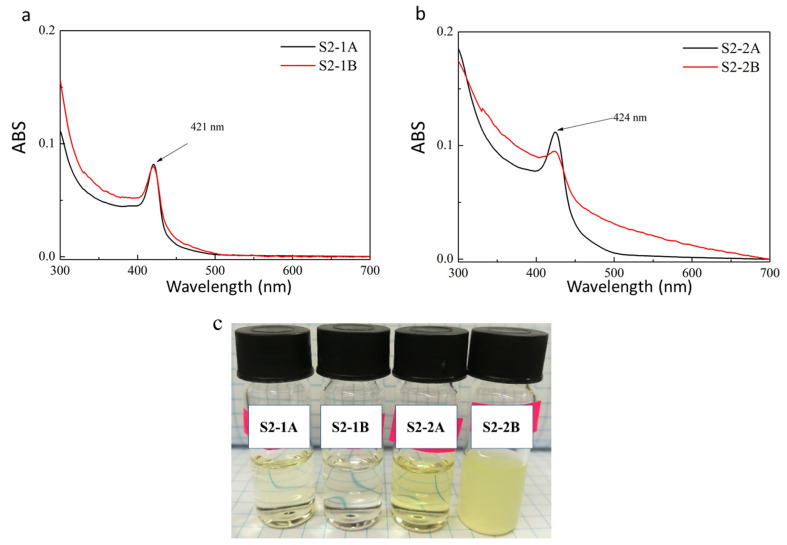
Absorbance spectra of S2 sample after forward LE reaction conducted by (**a**) 3-MPA; (**b**) DPA ligand. LE conducted under acidic (black line) and alkaline (red line) conditions; (**c**) Digital image of NPLs and NPs solution dispersed in PB.

**Figure 6:**
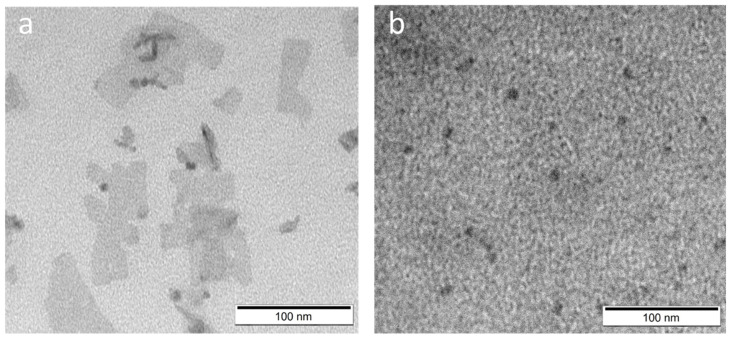
TEM pictures of S2 sample after forward phase transfer conducted by (**a**) 3-MPA; (**b**) DPA ligand. Both samples has been modified in acidic conditions.

ABS measurements were performed after forward phase transfer and normalized results are shown in Figure 5a,b for sample S2. The ABS peak derived from S2 NPLs at 408 nm was shifted to 421 nm after the 3-MPA surface modification (Figure 5a) and to 424 nm after the DPA modification (Figure 5b), regardless of the pH under which the forward phase transfer was performed. In this case, the forward phase transfer reaction also proceeded with much higher efficiency under acidic conditions. Based on the ABS results for the NPLs solution directly after forward phase transfer (without dilution), it was found that there were 5.2-times more nanoparticles in the S2-1A than in the S2-1B sample, and 4.7-times more NPLs in the S2-2A than in the S2-2B sample. The difference in NPLs concentration in PB solution is clearly evidenced as varied colour intensity, as presented in Figure 5c. As previously, TEM measurements were made for NPs prepared in acidic conditions: S2-1A (Figure 6a) and S2-2A (Figure 6b). Both samples underwent surface etching, although the obtained solutions showed greater homogeneity as compared to S1-1A and S1-2A. Sample S2-1A showed mainly flat structures, similar to the original S2 sample, however, with an apparent increase of NPLs lateral size, and with a predisposition to aggregate. This may prove that 3-MPA as a ligand incorporated into the structure of NPLs, creating an additional monolayer (421 nm corresponding to 2.9 eV, characterized by 7 monolayers) [14]. The constituents of the NPs surface can undergo exchange to a certain extent and during this process up to one new monolayer may be formed [15]. Thus, the difference to form a half-monolayer (S1 NPLs) or single-monolayer (S2 NPLs) in case of used 3-MPA could be a consequence of various surface condition of initial NPLs.

In the case of S1-2A sample, only spherical, non-aggregating nanoparticles (NPs) can be observed, with a size of approximately 10.9 nm in diameter. The photoluminescence of the obtained samples was also measured, however none of them showed emission.

As was described at the Materials and Methods section, reverse phase transfer, which allows to transfer NPLs from the aqueous phase back to the organic phase (toluene), were prepared for NPLs modified by 3-MPA (at pH = 4, S2-1A) and DPA (at pH = 4, S2-2A). For that purpose, the following combinations of ligands were used: Ac, OA, Cd (OL)_2_, Cd (Ac)_2_ and 3:1 Cd (OL)_2_:Cd (Ac)_2_ (Table 2). After modification by Ac and Cd (Ac)_2_ both S2-1A and S2-2A samples formed milky and cloudy solutions. Despite attempts to dissolve in organic and non-organic media, the sediments did not dissolve. Due to this, measurements of absorbance were impossible. Furthermore, no emission was observed after laser excitation of these samples. After modification by Cd (OL)_2_, clear and colourless solutions were obtained. However, ABS and PL measurements did not prove a presence of NPs. After modification by OA, clear and colourless solutions with small number of NPs with insignificant blue emission were obtained. In case of NPs surface modification using a mixture of Cd (OL)_2_:Cd (Ac)_2_ (in ratio 3:1), clear and colourless solutions with significant amount of NPs were received for S2-1A as well as for S2-2A samples. These samples are marked as R1 and R2, respectively, and both showed intensive blue emission.

**Figure 7:**
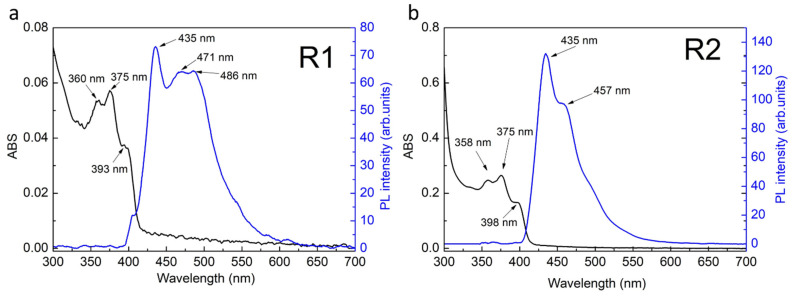
Absorbance and photoluminescence spectra after reverse phase transfer process for (**a**) R1 and (**b**) R2 sample.

**Figure 8:**
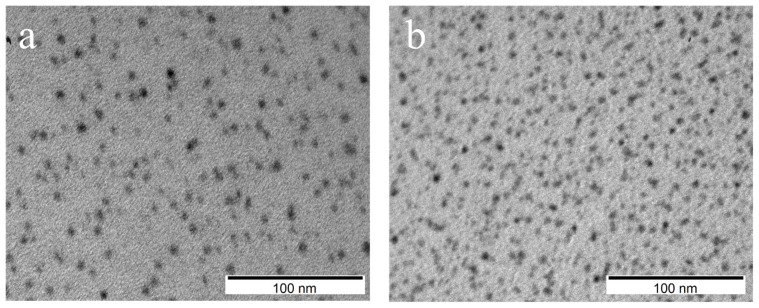
TEM image of (**a**) R1 and (**b**) R2 sample after reverse phase transfer.

**Figure 9:**
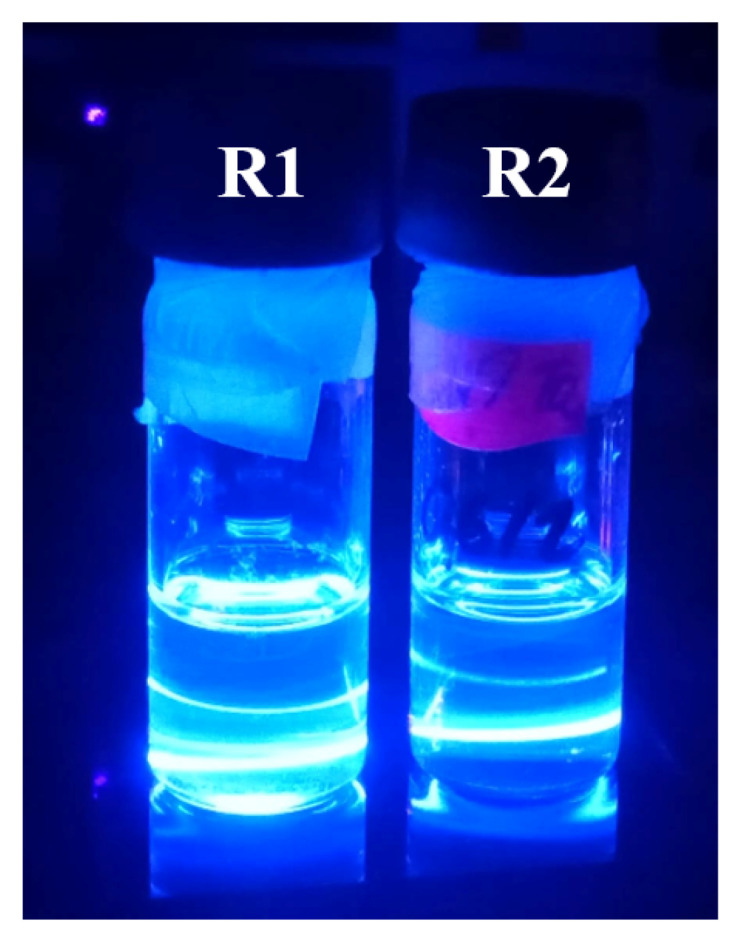
Digital image of NPs solution in toluene after reverse phase transfer excited by a laser.

After the reverse LE reaction, a series of ABS and PL measurements were made and the results are shown in Figure 7a,b. The single absorbance peak (about 420 nm) of the hydrophobic samples changed to several peaks placed at 360 nm, 375 nm and 393 nm for R1 and 358 nm, 375 nm and 398 nm for R2 sample. Photoluminescence of R1 sample showed the presence of distinct emission bands with a maximum intensity at 435 nm, with companion of wide band having two maxima at 471 nm and 486 nm. In the case of sample R2, a distinct band was observed in the photoluminescence spectrum with a maximum intensity at 435 nm and a faintly visible band at 457 nm. Based on these results, it was found that the samples were not homogeneous. For verification of nanoparticle morphology, TEM measurements were carried out, and their results are presented in Figure 8. The morphological distribution of R1 sample showed spherical structures with an average diameter of ~6 nm (Figure 8a). This means a significant shape transformation from flat NPLs to spherical NPs took places during reverse modification of R1 sample. The spherical shape of NPs was conserved for R2 sample, however their average size decreased (average diameter ~4 nm) as compared to initial S2-2A sample. The above results confirm that by applying consecutive forward and reverse LE reaction, nonemissive CdS NPLs can be transformed to blue emitting NPs having spherical shape. The intensive blue emission of R1 and R2 solutions are presented in Figure 9. Finally, to determine the efficiency of photoluminescence, the PL QY measurements were conducted and the results were 89% and 91% for R1 and R2 sample, respectively. It is possible that the defect-free NP surface obtained during consecutive forward and reverse phase transfer is responsible for such exceptional optical properties [16]. This value of PL QY is e.g., much higher than for most of the blue emitting organic fluorophores [17].

## 3. Materials and Methods

### 3.1. Materials

Cadmium oxide (CdO; 99.5%), oleic acid (OA; technical grade 90%), octadecene (ODE; technical grade 90%), zinc acetate (Zn(Ac)_2_; 99.99%), sulfur (S; 99.998%), d-penicillamine (DPA; 99%) and 3-mercaptopropionic acid (3-MPA; 99%) were purchased from Sigma-Aldrich (Saint Louis, MO, USA). Acetic acid (Ac, 99.7%), ethanol (99.8%), hexane (95%), hydrochloric acid (98.5%), sodium hydroxide (98.8%), potassium dihydrogen phosphate and disodium hydrogen phosphate were purchased from Chempur (Piekary Śląskie, Poland). All chemicals were used without further purification.

### 3.2. Synthesis of CdS Nanoplates

#### 3.2.1. Synthesis of CdS NPLs (S1)

CdS nanoplates were prepared based on the procedure described by Woznica et al. [12] with some changes. A total of 1 mL of Cd(OL)_2_ (0.4 M) (obtained by mixing CdO with OA and ODE in 250 °C), 1 mL of S-ODE (0.2 M), 18 mL of ODE and 37.7 mg (0.2 mmoL) of Zn(Ac)_2_ were mixed in a three-neck flask and degassed for 20 min in room temperature. Then the temperature was raised to 240 °C and the mixture was left for 60 min in argon flow until nanoplates were formed.

#### 3.2.2. Synthesis of CdS NPLs (S2)

The reaction was similar to the S1 synthesis, except the amount of Zn(Ac)_2_ was 73.4 mg (0.4 mmoL), and the synthesis temperature was set to 260 °C [12].

#### 3.2.3. Nanoplates Purification

The synthesis solution (for both samples) was mixed with oleic acid in proportion 2:1 and centrifuged at 6000 rpm (4430 rcf) for 15 min. The precipitate was redispersed in toluene and washed again with ethanol in proportion 1:2 (centrifugation at 6000 rpm for 5 min). The precipitate was then again dispersed in toluene.

### 3.3. Forward Phase Transfer

Before modification, all NPLs had hydrophobic ligands on the surface. Based on the modified literature procedure [9,10], eight reactions of functionalization of NPLs, half using DPA and half using 3-MPA, were prepared. Firstly, two kinds of aqueous phases were prepared as follows: DPA or 3-MPA (both 30.5 mg/mL) were added to phosphate buffer (PB, pH = 7.4) to prepare 10 mL of solutions each; these solutions were then divided into 8 equal samples (2.5 mL each) and each was adjusted to the appropriate pH (pH = 4 or 11) with 0.1 M HCl or 0.1 M NaOH. Then, 1 mL of NPLs solution in toluene was added to each of the eight previously prepared DPA and 3-MPA samples. The samples were shaken by orbital shaker for 24 h at room temperature. NPs were purified with ethanol:n-hexane (3:1) mixture using vortex and centrifugation (5 min × 6000 rpm). These operations were repeated by adding 1 mL of methanol until complete precipitation of NPLs from the solution was achieved. The supernatant was removed from the NPLs precipitate by decantation. Then, the precipitate was dissolved in 2 mL of phosphate buffer (pH = 7.4) and transferred into a vial through a 0.45 μm PTFE syringe filter. The complete list of samples prepared in forward phase transfer reaction is presented in Table 1 in the Results and Discussion section.

### 3.4. Reverse Phase Transfer

Reactions type reverse from the aqueous phase to the organic phase were prepared for NPs modified by 3-MPA (at pH = 4, sample S2-1A) and DPA (at pH=4, sample S2-2A) using the following combinations of ligands: Ac, OA, Cd(OL)_2_, Cd(Ac)_2_ and Cd(OL)_2_:Cd(Ac)_2_. All samples were prepared the same way and the exemplary procedure for Cd(OL)_2_ and Cd(Ac)_2_ mixture is described in detail below.

Firstly, the organic phase was prepared as follows: 75 mg of Cd(OL)_2_ and 25 mg of Cd(Ac)_2_ were dissolved in 4 mL of chloroform, then 0.25 mL of this solution was added to S2-1A and to S2-2A samples, each 1 mL in phosphate buffer. The samples were shaken by orbital shaker for 24 h at room temperature. NPs were purified with ethanol:n-hexane (2:1) mixture using vortex and centrifugation (10 min × 6000 rpm). These operations were repeated by adding 1 mL of methanol until complete precipitation of NPs from the solution was achieved. The supernatant was removed from the NPL precipitate by decantation. The samples were left overnight until the solvent was completely evaporated. Then, precipitates were dissolved in 2 mL of toluene. The received samples were labelled as R1 and R2 when S2-1A and S2-2A was modified, respectively.

### 3.5. Nanoparticle Characterization

Absorbance spectra (ABS) were measured on JASCO V-570 spectrophotometer. Photoluminescence (PL) was induced by 405 nm laser (CNI laser, 1 mW). The emission was collected with an optical fiber and PL spectra were recorded using CCD spectrometer (AvaSpec-ULS2048XL, Avantes, Louisville, CO, USA). Transmission electron microscopy (TEM) were performed on carbon-copper grids on the Hitachi H-800 microscope.

## 4. Conclusions

As a result of a NPLs surface modification carried out by a double ligand exchange protocol, involving forward and reverse phase transfer, nonemissive CdS NPLs were transformed to blue emitting NPs having spherical shape. These final NPs do not aggregate but form a stable colloidal solution in organic phase. Importantly, the NPs obtained according to the described protocol are characterized by efficient blue emission (PL QY = 89–91%), which is an exceptionally high value [18], especially considering that those NPs are only a core without an inorganic shell. The proposed method of NP synthesis and surface modification allows us to obtain blue emitters, which may find applications in e.g., diodes [19,20], QLED displays [21], or sensors [22].

## 5. Patents

Polish patent pending (P.437324 (of 17 March 2021), “Method of obtaining cadmium-based nanoparticles with high quantum efficiency”, A. Lesiak, H. Woźnica, M. Bański, A. Żak, J. Cabaj, A. Podhorodecki

## Figures and Tables

**Table 1:** Conditions of forward phase transfer and names of the samples.

Type of Nanomaterial	Name of NPs Sample after LE (Phosphate Buffer, pH = 7.4)	Ligand and pH Conditions of LE Reaction
S1–CdS NPLs hydrophobic in toluene	S1-1A	3-MPA, pH = 4
	S1-1B	3-MPA, pH = 11
	S1-2A	DPA, pH = 4
	S1-2B	DPA, pH = 11
S2–CdS NPLs hydrophobic in toluene	S2-1A	3-MPA, pH = 4
	S2-1B	3-MPA, pH = 11
	S2-2A	DPA, pH = 4
	S2-2B	DPA, pH = 11

**Table 2:** Summary of results after modification with various ligands during the reverse LE reaction.

Processed Samples	Ligands	Results	Name of the Sample after Reverse Modification
S2-1A S2-2A	Ac, Cd (Ac)_2_	Milky and cloudy solution, sparingly soluble both in organic and non-organic solvents	-
	OA	Small number of NPs, insignificant blue emission	-
	Cd (OL)_2_	Lack of NPs	-
	Cd (OL)_2_: Cd (Ac)_2_ (in ratio 3:1)	Significant number of NPs, blue emission	R1 (before S2-1A) R2 (before S2-2A)

## Data Availability

The data presented in this study are available on request from the corresponding author.
